# Treating Post-stroke Epilepsy in a Patient With Multiple Comorbidities

**DOI:** 10.7759/cureus.38483

**Published:** 2023-05-03

**Authors:** Marilena Mangiardi, Gianmarco Iaccarino, Michele Alessiani, Adriano Bonura, Sabrina Anticoli

**Affiliations:** 1 Stroke Unit, Azienda Ospedaliera San Camillo Forlanini, Rome, ITA; 2 Unit of Neurology, Neurophysiology, Neurobiology, Department of Medicine, Campus Bio-Medico University, Rome, ITA

**Keywords:** ischemic stroke, seizure, non valvular atrial fibrillation, post-stroke epilepsy, antiseizure medications

## Abstract

Stroke is a major cause of seizures and epilepsy in adults. Stroke severity, younger age, hemorrhagic subtype of stroke, and alcohol use have been identified as risk factors for the development of stroke-related epilepsy. Despite being a common complication in stroke survivors, current guidelines do not provide strong recommendations about the optimal treatment of post-stroke seizures. No clear guidance is given about the preferred antiseizure medications (ASMs), primary and secondary prophylaxis, and ASMs withdrawal. The management of older patients is further complicated by the presence of comorbidities, pharmacokinetic alterations, and intake of several medications. We present a case of a 77-year-old man affected by epidermolysis bullosa and diabetes mellitus, who suffered from ischemic stroke and then developed post-stroke seizures. This case shows how complex it is to manage post-stroke seizures in an older patient with multiple comorbidities.

## Introduction

Stroke is the most common cause of epilepsy in adults older than 35 years and more than half of newly diagnosed cases of epilepsy in elderly individuals are due to stroke [1,2]. Post-stroke seizures and epilepsy are classified as acute symptomatic (previously termed early-onset) when they occur within the first seven days after a stroke and as unprovoked seizures (also known as late-onset seizures) if they occur after the first week, with the first group more common than the second [2,3]. Several studies have found that the most common risk factors are young age (< 65 years), alcohol intake, hemorrhage, stroke severity, and cortical location of the stroke [4,5]. Due to the lack of randomized controlled trials (RCTs), existing guidelines provide only weak recommendations for the management of patients with post-stroke seizures [6,7]. Treatment of stroke-related epilepsy in patients with many diseases for which they take multiple drugs thus remains suboptimal.

## Case presentation

A 77-year-old patient with epidermolysis bullosa, under steroid treatment and diabetes mellitus, presented to the emergency department because he fell while riding his bicycle and lost consciousness.

**Figure 1:**
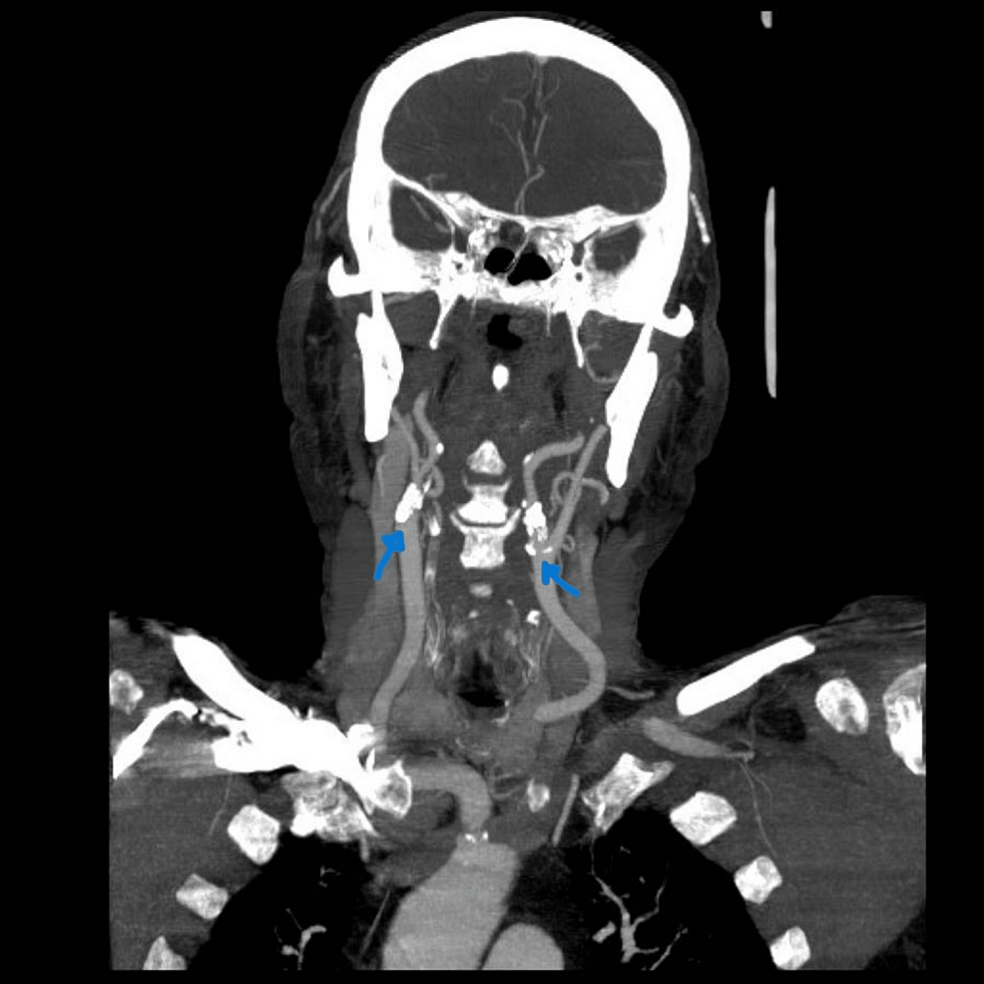
Computed tomography angiography (CTA) Tight stenosis in both internal carotid arteries

Because of the fall, he had trauma to his ribs and pelvis, so he was admitted to the internal medicine department. During his hospital stay, he abruptly developed a motor and sensory deficit in his left arm and leg, so he was transferred to the neurology department where a computed tomography angiography of the head and neck (CTA) was performed (Figure 1).

**Figure 2:**
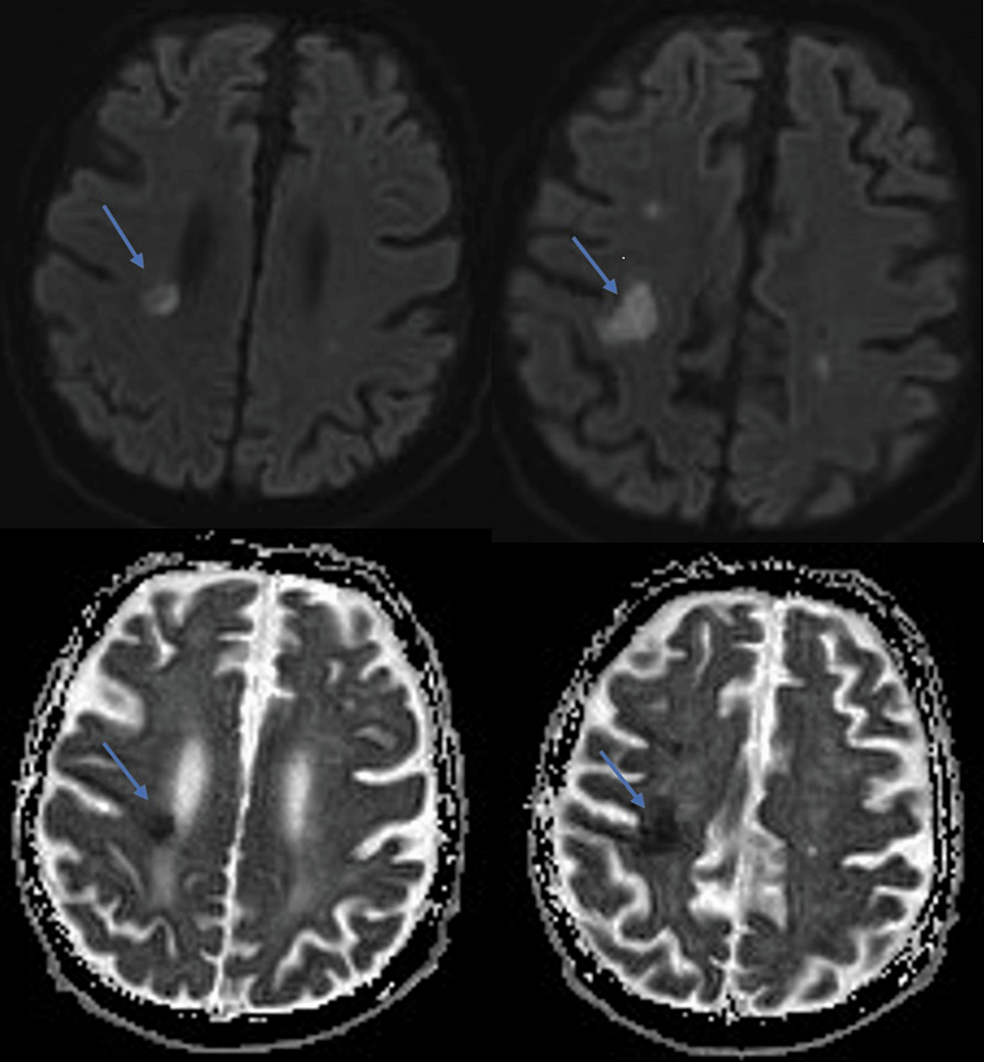
Brain MRI Both axial DWI images (on the top) and ADC map (on the bottom) showed a signal restriction in the right frontoparietal region. DWI: diffusion-weighted imaging; ADC: apparent diffusion coefficient

**Figure 3:**
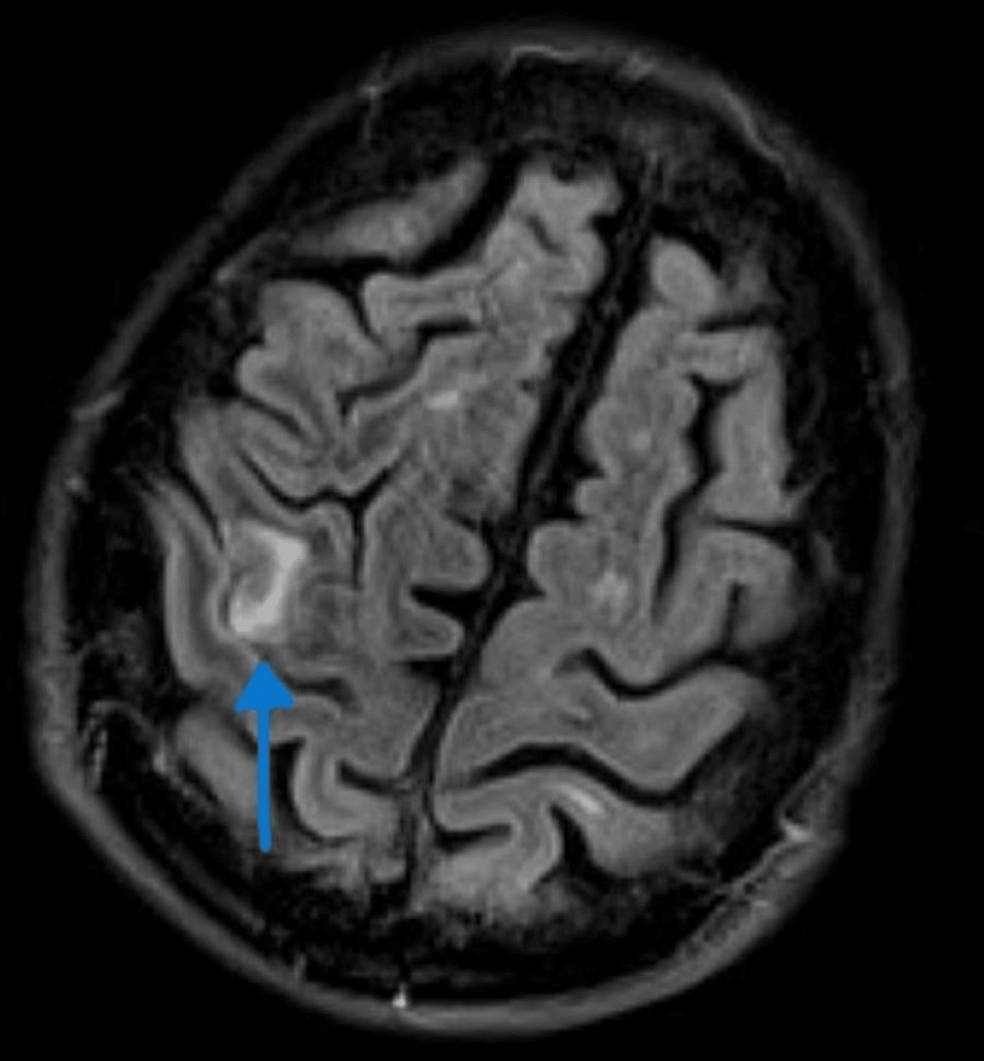
Brain MRI Axial T2 FLAIR weighted images showed an ischemic lesion in the right frontoparietal region. FLAIR: fluid-attenuated inversion recovery

Images showed tight stenosis (60-65%) in both internal carotid arteries. The brain MRI demonstrated the presence of an ischemic lesion in the right frontoparietal region with associated hemorrhage, thus confirming the diagnosis of ischemic stroke (Figures 2-3). Surgical intervention was not recommended by the vascular surgeons because of the short time interval since symptom onset. Instead, they suggested performing a stenting of the right internal carotid artery one month after the stroke onset.

**Figure 4:**
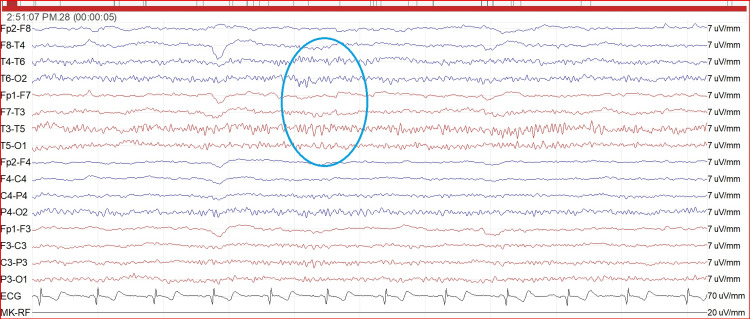
Standard electroencephalogram Intercritical EEG showed a slowing of basal activity and the presence of some slow-pointed waves in the frontotemporal regions bilaterally (blue circle).

A 24-hour recording of the heart rhythm revealed atrial fibrillation and an atrioventricular block. However, anticoagulant therapy could not be started straight away because of brain hemorrhage and because the skin blisters caused by epidermolysis bullosa represented a significant source of bleeding. In addition to that, left atrial appendage closure was not feasible due to the unsuitable heart anatomy, as demonstrated by a heart CT scan. Despite this, the patient was put on antiplatelet therapy due to the occurrence of an ischemic stroke. Later on during his hospital stay, he became febrile: blood cultures were positive for Staphylococcus (S.) aureus and S. haemolyticus infection, while the urine cultures were positive for Enterococcus (E.) faecalis infection. Adequate antibiotic therapy was thus started. Seven days after the stroke, the patient had one tonic/clonic seizure and many focal seizures without loss of awareness. The intercritical electroencephalogram showed a slowing of background activity and the presence of some slow-pointed anomalies (Figure 4).

Because of the atrioventricular block, anti-seizure therapy with lacosamide could not be prescribed. Levetiracetam was also not a suitable choice because of its interactions with direct-acting oral anticoagulants (DOACs). Antiseizure therapy was then initiated with brivaracetam, and the patient had no other seizures. At the end of the antibiotic treatment, he was discharged from the hospital. At a follow-up visit, when the skin blisters were no more bleeding, low-dose anticoagulant therapy was started in addition to the antiplatelet and antiseizure therapies.

## Discussion

Approximately 11% of individuals who suffered from stroke are at risk of developing post-stroke seizures or post-stroke epilepsy [7]. Several hypotheses have been put forward to explain the occurrence of seizures in these patients: ion channel alterations, altered metabolism, global hypoxia, and reperfusion damage. Regardless of the pathophysiologic mechanism, stroke-related seizures seem to occur more often in younger patients, in those who experienced a more severe stroke or a hemorrhagic stroke [8]. Their occurrence is also associated with a poorer prognosis and worse quality of life [9]. Epidermolysis bullosa (EB) is an inherited, heterogeneous group of rare genetic dermatoses characterized by mucocutaneous fragility and blister formation, inducible by often minimal trauma. A broad phenotypic spectrum has been described, with potentially severe extracutaneous manifestations, morbidity, and mortality. Over 30 subtypes are recognized, grouped into four major categories, based predominantly on the plane of cleavage within the skin and reflecting the underlying molecular abnormality: EB simplex, junctional EB, dystrophic EB, and Kindler EB. The study of EB has led to seminal advances in our understanding of cutaneous biology. To date, pathogenetic mutations in 16 distinct genes have been implicated in EB, encoding proteins influencing cellular integrity and adhesion. Precise diagnosis is reliant on correlating clinical and electron microscopic and immunohistological features with mutational analyses. In the absence of curative treatment, multidisciplinary care is targeted toward minimizing the risk of blister formation, wound care, symptom relief, and specific complications, the most feared of which - and also the leading cause of mortality - is squamous cell carcinoma. There is no known correlation between EB and epilepsy. Although our patient was 77 years old, he had a hemorrhagic transformation of the cerebral infarction and, furthermore, the hemorrhage was in the cortical region, another recognized risk factor for seizure development [9]. The management of such patients remains an open issue. Despite the high risk of recurrence (55-90% for unprovoked seizures), existing guidelines provide a weak recommendation against both primary and secondary prophylaxis due to the absence of RCTs [10]. Because our patient experienced multiple episodes of seizures during his hospital stay, we decided to start an antiseizure therapy. The choice of the most suitable drug was made difficult by the many comorbidities of our patient. Even though anticoagulation therapy could not be started as soon as atrial fibrillation was detected (brain imaging showed cerebral hemorrhage and epidermolysis bullosa caused bleeding skin blisters), we still needed to prescribe a DOAC after hemorrhage reabsorption and skin lesion healing. As a matter of fact, left atrial appendage closure, which was demonstrated to have a protective effect on thromboembolic events [11], was not a viable option because the CT scan showed unfavorable anatomy of the left atrial appendage. Only a few studies have compared the efficacy of ASMs in post-stroke seizures, and they show that, even though there is no significant difference in terms of efficacy, levetiracetam has a good tolerability profile [12]. However, pharmacokinetic studies have shown that levetiracetam and DOACs cannot be co-administered because levetiracetam is a P-gp inducer. In addition to that, we could not start therapy with lacosamide because the EKG demonstrated a first-degree atrioventricular block, and PR interval prolongation is a recognized side effect of lacosamide [13]. We thus decided to administer brivaracetam, a newly marketed ASM with a good safety and tolerability profile [14,15].

## Conclusions

This case shows how challenging it is to manage post-stroke seizures in real-world patients. As a matter of fact, even though young age is a known risk factor for the development of post-stroke seizures, stroke is by far more common in older individuals who have many comorbid conditions. The presence of comorbidities, the resulting intake of multiple medications, and the lack of guidelines make it difficult to choose the therapy and manage these patients.
